# Evaluation of an iPhone Otoscope in a Neurotrauma Clinic and as an Adjunct to Neurosurgical Education

**DOI:** 10.21767/2471-9633.10004

**Published:** 2016-01-29

**Authors:** Ronald Sahyouni, Omid Moshtaghi, Ramin Rajaii, Diem Kieu Tran, David Bustillo, Melissa Huang, Jefferson W Chen

**Affiliations:** 1 School of Medicine, University of California, Irvine, USA; 2 Division of Neurotrauma, Department of Neurological Surgery, University of California, Irvine, USA

**Keywords:** Otoscope, Cellscope, Education, Digital, iphone, Ear, Tympanic, Neurotrauma

## Abstract

**Introduction:**

CellScope^®^, an iPhone-enabled otoscope, was introduced into the neurotrauma clinic at an American College of Surgeons certified Level I trauma center. CellScope is an innovative tool that digitally improves optical clarity of the tympanic membrane, providing the acquisition of HIPPA compliant images. We compared the CellScope to the traditional otoscope in teaching medical students, neurosurgery physician assistants, and neurosurgery residents. In addition, the utility of this device in a neurotrauma clinic was specifically examined because of the high frequency of otologic symptoms after head trauma.

**Method:**

CellScope examination of the tympanic membranes was introduced as a standard/routine part of the exam of neurotrauma patients. We retrospectively reviewed the clinic charts of the NeuroTrauma patients during a three-month time period to determine if their otologic symptoms correlated with any CellScope visualized abnormalities. Medical students, P.A.s, residents, and attendings were surveyed before and after using CellScope to assess their comfort and skill in completing an otological exam, as well as their opinion on the utility of CellScope in their medical training.

**Results:**

18 medical professionals were surveyed before and after the use of CellScope. Surveys were graded on a 1-5 scale and indicated a greater preference for the CellScope (4.7/5.0) versus the otoscope (3.16/5.0). Similarly, there was a preference for the CellScope for medical education (4.7/5.0 versus 2.78/5.0). Finally, surveys showed a greater preference for CellScope in identifying abnormal pathology. The overall score showed a 49% increased preference for CellScope over the traditional otoscope. Six previously undiagnosed abnormalities of the tympanic membrane were identified in a total of 27 neurotrauma patients using CellScope.

**Conclusion:**

The visualization of the tympanic membrane is an important part of the physical examination of the neurotrauma patient. Smartphone-enabled medical instruments like CellScope may facilitate and remove barriers to routine implementation of this part of the physical examination.

## Introduction

**Figure 1:**
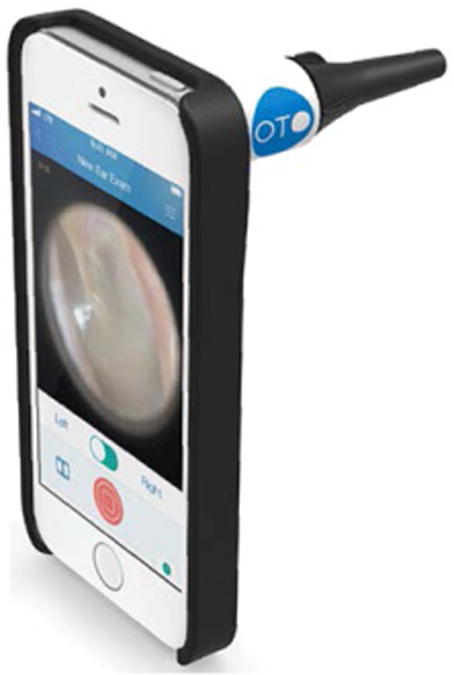
CellScope^®^: Attached to an iPhone 6 with disposable speculum displaying the tympanic membrane.

The routine use of the conventional otoscope is difficult not only because of its expense, but also because of the difficulty in visualizing the tympanic membrane anatomy through a small canal. As a result, otoscopes are used less frequently and thus medical students are receiving decreased exposure to this invaluable tool. Recent studies have demonstrated that both attending and resident physicians frequently have difficulties recognizing abnormal pathology of the middle ear [1, 2]. Using ear simulators and video otoscopy, educators have attempted to improve medical education of this difficult skill [3, 4]. In this climate, the need for tools that enhance the teaching of medical professionals is ever-present. Recently, CellScope^®^ (Figure 1), an iPhone-based otoscope, has emerged to address this issue. CellScope is an innovative tool that digitally improves the optical clarity of the tympanic membrane (TM) as viewed through the iPhone. This technology provides to medical professionals, both experienced and in-training, an enhanced view of the middle ear. Additionally, the fidelity of these images allows for open discussion among residents and attendings, providing diagnostic and educational value beyond that of a conventional otoscope. Furthermore, CellScope gives patients and clinicians of all levels the power to digitally record images, thus opening the door to remote consultations and follow up. Thus, access to medical care can be expanded to patients lacking access to health care due to geographic or logistical challenges. Currently, CellScope has only been reviewed and critically evaluated in two publications – both of which are focused on the pediatric population [5, 6]. This study took a different approach and incorporated CellScope in a neurotrauma clinic at an American College of Surgeons Verified Level I trauma center, where the routine use of CellScope and assessment of the TM is warranted as a result of traumatic brain injuries. These patients are more likely to have vestibuloauditory complaints that go along with their traumatic brain injuries and concussion (i.e., dizziness, tinnitus, hearing loss). The most common findings include hemotympanum or middle ear injury, which is seen in one-third of patients with severe head trauma and one-half of patients with temporal bone skull fractures [7-9]. In addition, middle ear injuries can occur after direct or penetrating trauma to the ear [10-14].

The purpose of this study is to determine the utility of CellScope in a neurotrauma clinic and in medical education. Three specific aims were targeted for this purpose: 1) to assess the value of routine TM visualization in a neurotrauma clinic: 2) to determine if CellScope enhances visualization of the TM compared to a conventional otoscope; and 3) to assess whether CellScope enhances communication and explanation of normal or abnormal pathology to colleagues and attending physicians. In addition, two secondary aims served to supplement our study: 4) to assess whether CellScope enhances the ability of the attending physician to teach medical students and residents pertinent topics related to otoscopic evaluation of patients and 5) to assess the efficacy of CellScope in assisting medical personnel in medical diagnosis of middle ear pathology. We hypothesized that the use of CellScope will enrich and enhance the medical education of medical personnel when compared to a traditional otoscope.

## Methods

**Figure 2:**
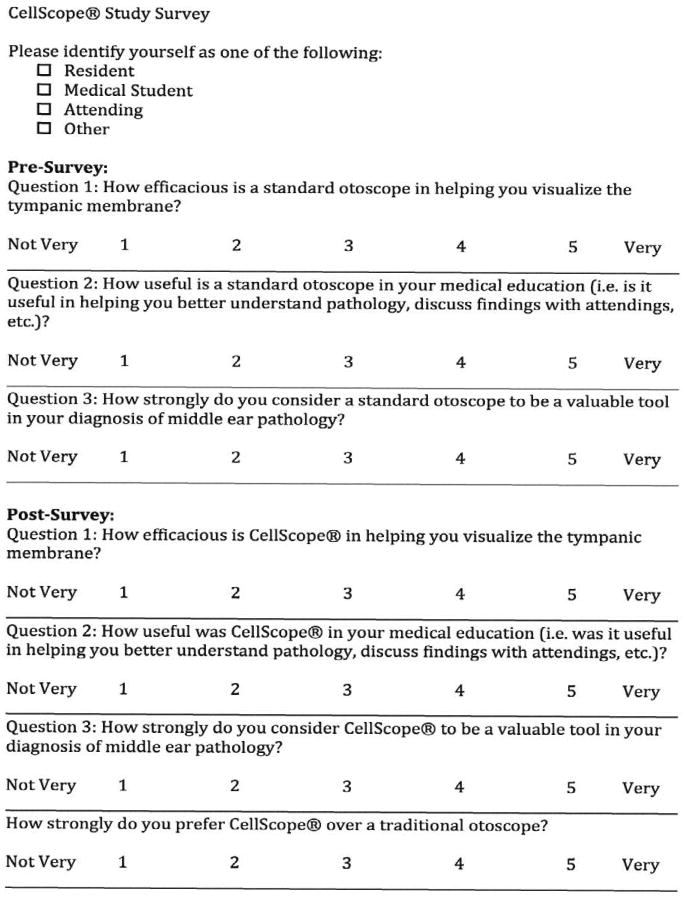
Survey used for medical practitioners.

CellScope is an FDA-exempt class 1 device due to the insignificant risk to the patient and has been approved for use as a smartphone-enabled otoscope [5, 6]. The CellScope attachment for the iPhone was obtained from CellScope Inc. (San Francisco, California). Our study endpoints consisted of pre and post-surveys (Figure 2) that were administered to all medical students, physicians' assistants (PAs), resident physicians, attending physicians, and a registered nurse who participated in the study of three month duration.

CellScope was implemented in the Neurotrauma Clinic operated by the Department of Neurosurgery at the University of California, Irvine Medical Center (UCIMC). This clinic is devoted to patients that have suffered a brain or spinal injury and include patients that have been seen in the UCIMC trauma center or have been referred from elsewhere for an opinion. The clinic is staffed by medical students, neurosurgery residents, neurosurgery PAs, neurosurgery R.N.s and neurosurgery attending. All patients in this clinic are seen by an attending neurosurgeon. Use of CellScope did not cause any deviation from normal patient history or physical examination procedures. After all remaining patient questions were answered, and upon conclusion of the patient interaction, the patient was asked to consent to having their tympanic membrane visualized and photographed with CellScope. They were informed that the photographs taken would have no personal information attached and were for teaching purposes. Upon consent, CellScope was used on an iPhone 5 with the appropriate case-attachment provided by the company. It was used in the same manner as a traditional otoscope, and advanced into the external auditory canal of the patient, until visualization of the TM was achieved. At that point, several photos were taken and the process was repeated on the contralateral ear. Each image was stored via secure, encrypted, HIPAA-compliant software provided with the CellScope application.

## Results

### Utility in a neuroTrauma clinic

**Figure 3:**
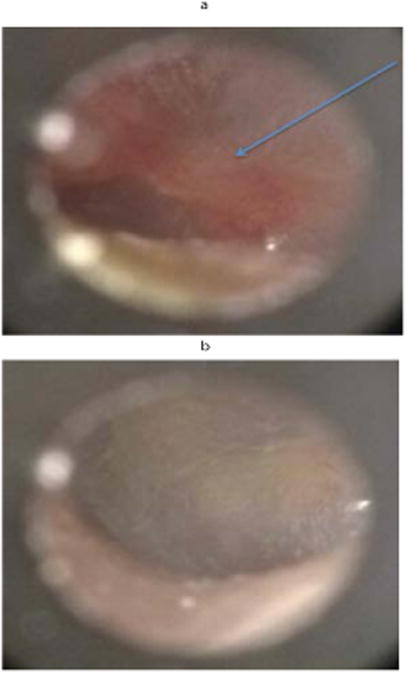
a)Patient presenting to neurotrauma clinic with dizziness, difficulty walking, and ear pain following head trauma. Upon visualization of TM hemotympanum diagnosis was confirmed. Arrow pointing to blood located medial to TM. b)Same patient's contralateral ear was examined showing no pathology.

CellScope was used to examine the tympanic membranes of 27 neurotrauma patients (Charts 1 and 2) in a Neurotrauma clinic over a three-month timeframe. Six patients (22%) displayed an ear-related abnormality that could be visualized via CellScope. These cases included hemotympanum, eardrum perforation, otitis media, external auditory canal laceration, and scarring of the tympanic membrane. Figure 3 is an example of the findings in a patient with hemotympanum from a basilar skull fracture. Although the majority of the patients seen in this clinic had a primary diagnosis of traumatic brain injury (TBI), there were a significant number (14.8%) in the “other” category who was largely referred patients with an unclear history of TBI or syncope. In total, there were 12/27 (44%) patients that had some symptoms of hearing loss or dizziness that would warrant otologic examination. Of the patients with a primary diagnosis of TBI (n=19), there were 7 that had these symptoms (37%).

### Efficacy as a teaching tool

Eighteen subjects, including neurosurgery residents (n=5), medical students (n=5), physician assistants (n=5), a registered nurse (n=1), and attending neurosurgeons (n=2) used CellScope and completed the surveys. Answers from the pre- and post-survey were scaled from 1 to 5, with the higher score indicating a more favorable rating. The pre-survey questions assess the efficacy, usefulness, and value of the standard otoscope while the post-survey questions examined the same parameters for the CellScope.

The surveys given were utilized to compare CellScope to the conventional otoscope. The average scores for each of the three pre- and post-survey questions were compared using a t-test (p<0.0001). A statistically significant difference is observed. As shown in Graph 1, the average post-survey question score is significantly greater than the average pre-survey question score. The total average score for the post-survey questions is 4.64 out of 5 compared to the 3.11 total average score for the pre-survey questions (49% increase).

#### Graph 1

The average score for the fourth question on the post-survey, which asked whether one preferred the CellScope over the traditional otoscope was 4.61 out of 5.0.

#### Graph 2

We also stratified the survey results by level of training, creating categories for attending physicians (n=2), physician assistants (n=5), medical students (n=5), resident physicians (n=5), and registered nurse (n=1).. All of these surveyed had previous formal training in the use of the otoscope and many had used it for many years in their practices. Graph 2 summarizes the average total scores for pre- and post- survey question, and further breaks it down by personnel title. The results showed that medical students had the greatest preference for the CellScope over the traditional otoscope regarding visualization, education, and diagnostics, out of all of the groups. The PA group showed the slightest preference for the CellScope. However, all of the groups did not have a significant variability in response for overall preference, with all groups agreeing that CellScope is a better choice.

## Discussion

Our study, though limited in sample size, confirmed our hypothesis relating to the efficacy and convenience of CellScope. CellScope was the preferred instrument over the traditional otoscope for all of the categories surveyed and for medical providers in all levels of training. CellScope is the favored tool in visualizing the tympanic membrane and can aid as a diagnostic tool equivalent or superior to the conventional otoscope. Furthermore, CellScope was preferred as a medical education tool in an anatomically difficult to visualize part of the body. Because it can display a magnified image onto a larger, viewable screen, it is very useful in helping others understand pathology and being able to discuss findings. In addition, although the patients were not formally surveyed, they consistently expressed preference for being examined by CellScope largely because of the opportunity to see images of their tympanic membrane. Furthermore, the large number of TMs that can be documented can be useful both in office and in a lecture setting to advance medical knowledge and diagnostic skill. In this respect, medical students and residents appear to benefit most from the CellScope. Finally, the CellScope was preferred as a valuable tool in diagnosing middle ear pathology. During standard exams, the CellScope can be used to check the patient's ear and potentially screen for any abnormalities. Thus, due to its clear resolution and ability to project and save images onto a screen, the CellScope is an innovative alternative that is favored over the traditional otoscope amongst our students and practitioners.

Throughout the course of this study, we found that the successful incorporation of CellScope in the evaluation of patients with traumatic brain injury (TBI) was feasible and practical. Given the utility, accessibility, portability, and effectiveness of CellScope as a diagnostic and educational tool, otoscopic examination of patients with TBI in a neurotrauma clinic is readily warranted. Some of the barriers that prevent consistent otoscopic examination in neurotrauma clinics include the difficulty of properly using a traditional otoscope, the lack of availability of traditional otoscopes in many neurotrauma clinics, the cost of a wall-mounted otoscope, and the inadequacy of TM visualization via traditional otoscopic examination. These barriers are effectively addressed by CellScope, making it a useful diagnostic and educational tool in the evaluation of TBI patients in neurotrauma clinics. One possible algorithm that can be used in order to determine whether CellScope should be employed is diagrammed below. Patients can be stratified by the presence of TBI, followed by the presence of vestibuloauditory symptoms (i.e. dizziness, tinnitus, hearing loss, etc.).

The use of smartphone-enabled otoscopes, such as the CellScope, has the potential to improve patient care. Its ease of use makes documentation of the TM not only easier for the clinician, but also sharable to the patient. The practice of telemedicine using a video otoscope has already proved advantageous [15-17]. CellScope expands upon this practice and makes use of digital otoscopes more accessible. Improving simplicity and accessibility of TM documentation allows for an expansion of current practices. For example, mid-level providers or residents can take a digital photograph of a tympanic membrane, and instantaneously send it to an expert for a second opinion. This has already been proven to be effective with video otoscopy, yet CellScope can do the same task with less equipment and training [18]. Additionally, our study suggests that CellScope improves visualization of the middle ear compared to conventional otoscopes. The perception, accuracy, resolution, and ease by which CellScope can visualize the TM are greatly improved with this technology. There is a clear increase in the subjective utility and preference of CellScope by the clinicians who participated in this study due to the many advantages and practicalities offered.

## Conclusion

CellScope visualization of TMs is a useful tool in a neurotrauma clinic. CellScope was preferred by medical providers of all stages of training, including residents, attendings, physician assistants, medical students, and registered nurses, for the examination of the outer and middle ear at UCIMC. Additionally, from the perspective of attending physicians, CellScope is a beneficial tool that can be used to educate medical residents. Moreover, the technological nature of CellScope allows continual technological updates, high-resolution and clear images, instantaneous transmission among colleagues, and remote examination of patients with limited clinic access. Finally, CellScope allows the magnification and expansion of images for improved education of medical and nursing trainees. Further studies need to be conducted to examine the benefits of CellScope - studies with a greater number of medical professionals and at a variety of different healthcare settings.

## Figures and Tables

**Figure:**
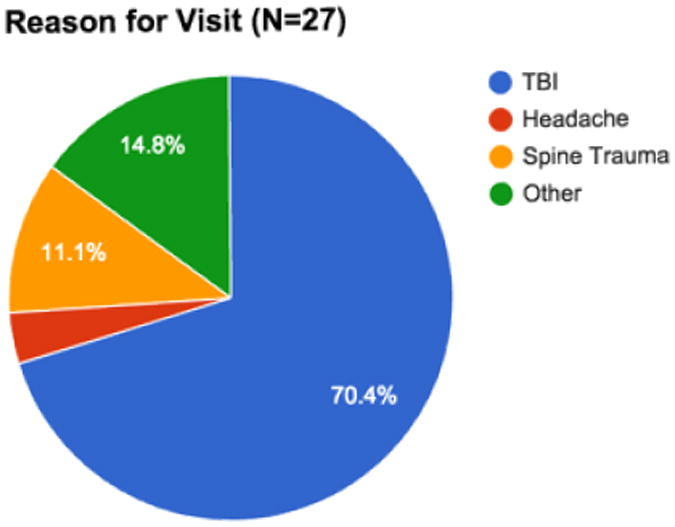
Chart 1 The principle diagnosis of the patients seen in the NeuroTrauma clinic.

**Figure:**
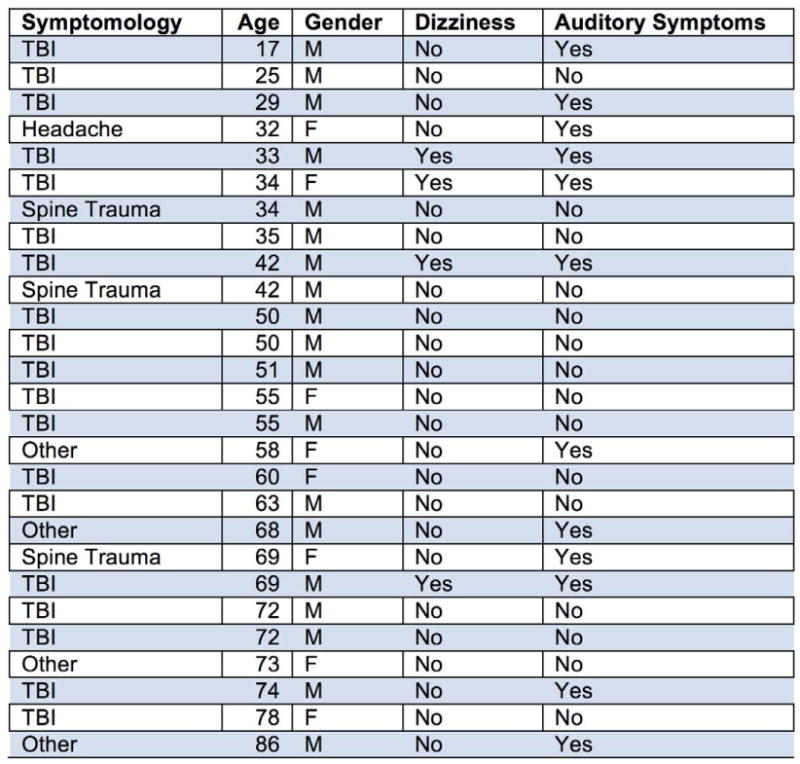
Chart 2 The demographics of the 27 patients presenting to the NeuroTrauma clinic.

**Figure:**
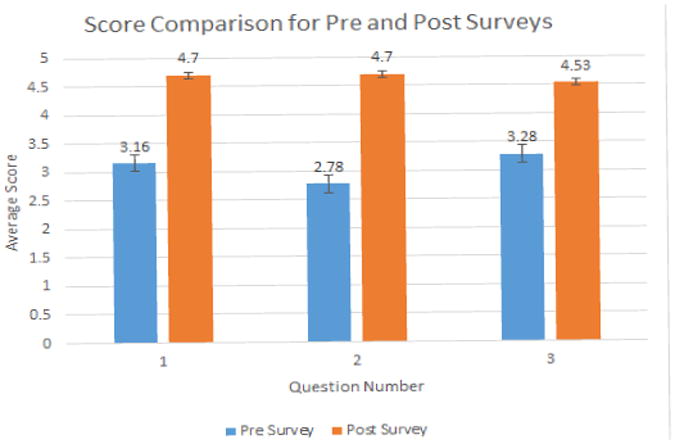
Graph 1 The representation of the pooled responses before and after the single use of the CellScope.

**Figure:**
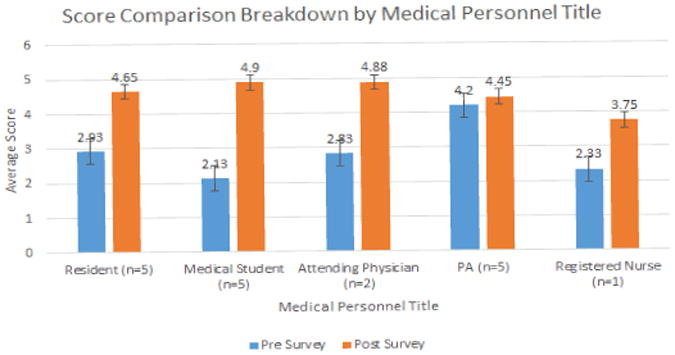
Graph 2 Survey breakdown according to the category of health provider.

**Figure:**
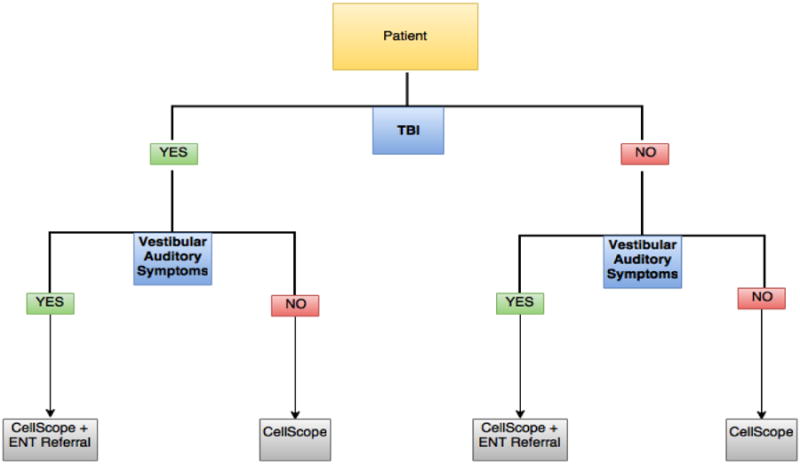
Flow chart A proposal for the integration of CellScope in the screening of TBI patients with symptoms that may possibly be reflective of pathology of the middle ear.

## Abbreviations

TBI: Traumatic Brain Injury
PA: Physician Assistant
HIPPA: Health Insurance Portability and Accountability Act
TM: Tympanic Membrane
UCIMC: University of California Irvine Medical Center
